# Potential mechanisms and modulators of food intake during pregnancy

**DOI:** 10.3389/fnut.2023.1032430

**Published:** 2023-01-20

**Authors:** Theresa Waclawek, Soyoung Q. Park

**Affiliations:** ^1^Berlin School of Mind and Brain, Humboldt-Universität zu Berlin, Berlin, Germany; ^2^Department of Decision Neuroscience and Nutrition, German Institute of Human Nutrition (DIfE), Potsdam, Germany; ^3^Charité–Universitätsmedizin Berlin, Neuroscience Research Center, Berlin Institute of Health, Corporate Member of Freie Universität Berlin, Humboldt-Universität zu Berlin, Berlin, Germany; ^4^Deutsches Zentrum für Diabetesforschung, Neuherberg, Germany

**Keywords:** nutrition, pregnancy, reward processing, cognition, hormones, metabolism, diet

## Abstract

Dietary choice during pregnancy is crucial not only for fetal development, but also for long-term health outcomes of both mother and child. During pregnancy, dramatic changes in endocrine, cognitive, and reward systems have been shown to take place. Interestingly, in different contexts, many of these mechanisms play a key role in guiding food intake. Here, we review how food intake may be impacted as a function of pregnancy-induced changes across species. We first summarize changes in endocrine and metabolic signaling in the course of pregnancy. Then, we show how these may be related to cognitive function and reward processing in humans. Finally, we link these to potential drivers of change in eating behavior throughout the course of pregnancy.

## Introduction

Pregnancy is a time of major hormonal, physiological, and cognitive change for the mother, and of vital development for the child. Dietary intake is particularly important during this period, as it shapes both short- and long-term health outcomes of mother and child. Diet during pregnancy influences the development of gestational disease in pregnant women (1). For example, it impacts gestational diabetes mellitus (2), which is the development of glucose intolerance during pregnancy (3), and pre-eclampsia (4, 5), which is the development of hypertension and increased protein levels in the urine during pregnancy (6). Gestational diabetes increases risk for hypertensive disorders (including pre-eclampsia) as well as preterm birth and infants born large for gestational age (7). Dietary intake during pregnancy also influences health outcomes through its impact on weight gain. Overweight and obesity, as well as excessive weight gain are associated with health complications during pregnancy (8), such as thrombosis (9) and caesarian delivery (9). In offspring, maternal food intake can impact neurobiological development. For example, maternal high fat diets influence dopaminergic (10), hypothalamic (11), and hippocampal (12) development in rodents. Maternal diet also impacts other important aspects of development, such as the infant’s gut microbiome (13).

Dietary choice during pregnancy continues to impact health outcomes of mother and child even after pregnancy. In offspring, nutrient exposure during pregnancy impacts disease development later in life (14) such as obesity, diabetes (15), cancer (16), and asthma (17). Higher diet quality during pregnancy has been associated with higher neurodevelopment (18) and intelligence scores (19) in childhood. Higher intake of highly processed foods in pregnancy has been associated with worse verbal functioning in childhood (20). The aforementioned impact of maternal high fat diets on neuronal circuitry development impacts eating behaviors later in life, for example, non-human primates exposed to such diets *in utero* are more likely to later choose foods high in fat and sugar, and also show suppressed dopamine signaling (21). The impact of maternal diet on the infant gut microbiome has important implications for health outcomes such as asthma (22) and the functioning of the immune system (23). In pregnant women, gestational diabetes is associated with at least a sevenfold increased risk of developing type 2 diabetes later in life (24), as well as an increased risk of cardiovascular disease (25). Preeclampsia is associated with a multitude of long-term health outcomes (26), such as roughly double the risk of early cardiac disease (27) and an increased risk of renal disease (28). Further, women who gain more weight during pregnancy retain more of this weight gain both 1 and 15 years after delivery (29). Diet during pregnancy, therefore, is important to the health of mother and child both during pregnancy and post-pregnancy.

Beyond the importance of preventing undesirable health outcomes, the magnitude of an event such as pregnancy may make it a “teachable moment.” A teachable moment is a life event during which those experiencing it are especially amenable to positive lifestyle behavioral change (30). Pregnancy can be considered such an event, as it is a period in which women are more concerned about health-related behaviors, and have increased contact with healthcare providers (31). Therefore, effective nutrition interventions may be especially impactful on positive long-term health behaviors of women if they are administered during pregnancy (31).

Despite the importance of dietary intake during pregnancy, sufficient research on how to improve diet and associated health outcomes during pregnancy is lacking. According to a review by Skouteris et al. (32), diet improvement outcomes from health interventions in pregnant women have produced inconsistent results. Encouraging healthy gestational weight gain through current healthcare provider advice has also not produced consistent improvements (33). Further, interventions are still not effective at improving many critical outcomes, such as gestational diabetes (1). There is a need to move beyond simple dietary advice, and incorporate other important factors guiding food intake (32). For this, we require a better understanding of the relevant mechanisms guiding dietary choice during pregnancy (32).

**FIGURE 1:**
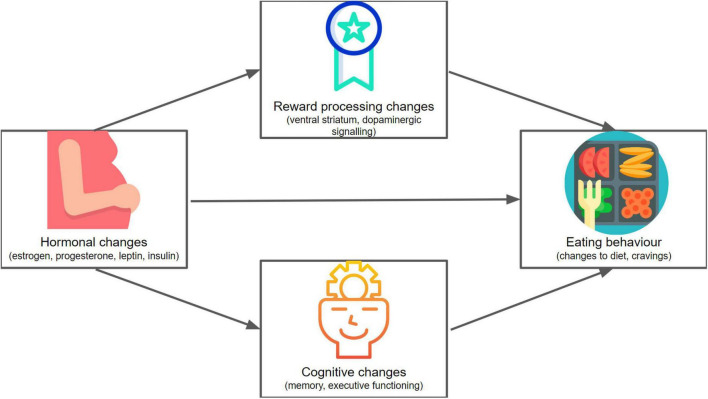
A conceptual mapping of the framework of this review. We explore hormonal (including metabolic), reward-related, and cognitive changes in pregnancy, and consider how these might affect eating behavior. Icons made by Freepik (136, 137), Icongeek26 (138) and catkuro (135) from www.flaticon.com.

Recent research on dietary choice has highlighted the importance of underlying mechanisms involving metabolic, reward, and cognitive processes (34). During pregnancy, the maternal body and brain undergo hormonally driven changes that result in alterations in these mechanisms of metabolic functioning (35), reward processing (36), and cognition (36). A better understanding of these pregnancy-related changes to important mechanisms underlying eating behavior would be helpful in understanding what shapes dietary choice during pregnancy (see Figure 1). This can foster the efficacy of healthcare provider advice and interventions to promote healthy dietary choice during pregnancy.

The aim of this narrative review is to better understand the physiological and cognitive mechanisms shaping dietary decision-making during pregnancy, and is structured as follows: first, we review how eating behavior changes during pregnancy. Then, we review the current understanding of pregnancy-related hormonal, metabolic, reward-related, and cognitive changes. Further, we review how these mechanisms can impact eating behaviors and food intake in general. Finally, we link these mechanisms to eating behavior during pregnancy. This review, therefore, will highlight an underexplored and important research direction involving the impact of pregnancy-induced changes on the eating behavior of pregnant women. Though the focus of this review is pregnancy, we occasionally draw upon postpartum research in areas in which research in pregnancy is limited and the postpartum findings can help us to better understand the pregnancy transition. Additionally, as described above, maternal nutrition has important consequences for offspring-related outcomes. Findings from this area of research, however, are largely beyond the scope of this review.

## Food intake during pregnancy

During pregnancy, total energy consumed increases (37). Specifically, resting metabolic rate can increase by about 29%, whereas energy intake can increase by about 9%, and fat mass can increase by around 4.5 kg when comparing pregnancy to pre-pregnancy (38). Self-reported food-intake of pregnant women seems to shift toward more healthy nutrition, as significant increases are observed in the consumption of fruit and vegetables, and decreases in the consumption of eggs, fried and fast foods, and coffee and tea (37).

Such a shift in nutrition seems to partly reflect the reported motivations to adjust diet in pregnancy, including the desire to optimize health outcomes for the fetus, to optimize nutrient intake, to enhance health, to lessen illness or to help pregnancy-induced nausea, as well as to satisfy craving and for enjoyment (39). Craving, in particular, is an often reported important motivator of food intake during pregnancy, but it is not yet determined what underlies reported increases in cravings during pregnancy (40) [although higher stress and worse sleep quality during pregnancy can exacerbate them (41)]. The most frequently reported changes in diet were in line with direction received by expectant mothers, such as to reduce caffeine consumption, to be careful in terms of food preparation, and to increase intake of both dairy as well as fruits and vegetables (39). Whereas advice to increase the intake of fish, meat, and alternatives are less well-followed, the motivation to reduce intake of harmful foods was more often reported than the motivation to increase intake of foods containing important nutrients, which leaves room for improvement in terms of dietary choice during pregnancy (39).

It is important to keep in mind that the research on dietary intake during pregnancy so far has primarily relied on self-reports (37). Although self-reported motivations for dietary choice can be informative, it is essential to know that these might deviate from actual food intake, and it is also important to understand how pregnancy itself might impact dietary choice. This challenge highlights the importance of understanding objectively observable factors, such as hormonal, reward, and cognitive mechanisms, since these guide eating behaviors.

## Hormonal signaling

### Major hormonal changes in pregnancy and food intake

Dramatic hormonal changes orchestrate the maternal adaptations necessary to meet the demands of a successful pregnancy (42). Progesterone levels, which normally decrease during the menstrual cycle, remain high and increase in response to the initial pregnancy signaling hormone human chorionic gonadotropin (hCG), and estradiol (an estrogen) levels increase during the second and third trimester (42). In the course of pregnancy, both estrogen and progesterone levels reach levels many fold higher than at any point during the menstrual cycle (43). Other important hormonal changes during pregnancy include those affecting glucocorticoids [which reach levels three times higher than usual by the third trimester (44)] as well as prolactin and growth hormone (42).

Interestingly, hormones such as estrogen, progesterone, prolactin, growth hormone, and cortisol have been reported to impact eating behavior in general. In non-pregnant populations, estrogen generally reduces food intake (45), protecting against binge eating (46), and has been a candidate hormone for treating obesity (47). In non-pregnant rodent models, estradiol administration can reduce weight gain (48, 49) and restore leptin sensitivity [a satiety-signaling hormone (48)], while estrogen depletion can lead to an increase in body fat (50). However, in pregnant populations, these estrogenic effects disappear. For example, thermogenesis of brown adipose (fat) tissue resulting from estradiol administration in non-pregnant rats is absent in pregnant rats (51).

The increased food intake and weight gain occurring alongside large increases in estrogen (35) during pregnancy suggest interactions with other pregnancy-related hormonal changes, such as an estrogen-progesterone interaction. Progesterone has been shown to influence dietary intake indirectly, for example through counteracting the effects of estrogen (35, 52). In non-pregnant populations, higher progesterone levels can increase the risk for maladaptive eating behaviors by reducing the protective effects of estrogen on, for example, binge eating behavior (46). It is the interaction of high levels of both progesterone and estrogen that has been associated with increased emotional eating across the menstrual cycle, rather than the independent effects of either (53).

In pregnant rodent models, prolactin interacts with satiety signaling by contributing to leptin insensitivity to promote food consumption (54), and growth hormone affects plasma glucose regulation and fat gain in pregnancy (35). Excessive cortisol production has been linked to increased fat accumulation in non-pregnant populations, as well as to metabolic disorders such as diabetes (55), but the role of cortisol in eating behavior during pregnancy remains unknown (35). Although many of these pregnancy-related hormones, as well as their complex interactions, point to their potential role on dietary intake and metabolic dynamics, these are largely under-investigated and present an important avenue for further research, especially since they dramatically change during pregnancy.

### Metabolic modulations during pregnancy and food intake

Energy homeostasis signaling is affected by the above-described hormonal changes in a manner that ensures adequate energy for fetal development and lactation (35). Here, we focus on the ingestive hormonal changes of leptin and insulin. Leptin is a hormone released from adipose tissue that maintains fat tissue levels by signaling the body’s current energy state and suppressing further food intake (56), and insulin is a hormone involved in facilitating muscle and fat tissue glucose uptake (57).

Pregnant women display both increased serum leptin levels (58) and decreased leptin sensitivity (59). This increase in leptin levels may result from an increase in fat mass during pregnancy (60), additional leptin secretion from the placenta (61), and a slower clearing of leptin from the blood during pregnancy (62). The functional role of increased leptin levels (beyond being a by-product of increased fat stores) is not fully known (54), but it plays a role in fetal development (63). Since leptin signals a reduction in the need for food intake, a decrease in leptin sensitivity (possibly due to the effect of prolactin and growth hormone) counteracts this and allows for the important maternal adaptation of greater food intake and positive energy levels (54).

Like leptin, insulin functioning has been shown to be altered during pregnancy *via* higher insulin levels on the one hand and decreased insulin sensitivity on the other (64). These changes in insulin functioning are thought to play an important role in ensuring adequate energy supply to the fetus through their role in glucose regulation (42), which is to facilitate glucose uptake in fat and muscle tissue (65). Glucose is transmitted to the fetus passively; for this to be possible, the mother’s glucose levels must remain higher than that of the fetus’, and so the mother’s tissues become less insulin sensitive (66). The fetus must also, however, be protected from excessive glucose exposure following a meal, and so higher levels of insulin are released to protect the fetus from overexposure due to the mother’s insulin-insensitive tissue (54). The changes in insulin during pregnancy have been associated with pregnancy-related weight gain (67). These changes both in insulin and leptin are conducive to creating a positive energy balance, which is thought to be necessary to provide energy to the growing fetus (68). As noted by Grattan and Ladyman (42), the pregnant body is engineered for weight gain, but this increases the risk for excessive gestational weight gain in our current food environment (42).

### Effects of hormonal modulations on reward processing and cognition

Receptors for hormones such as estradiol (69), progesterone (70), glucocorticoids (71), leptin (72), and insulin (73) are present throughout the brain, and have been shown to impact both reward processing and cognition (42, 74, 75). For example, the rewarding value of pup stimuli in rodents is mediated by progesterone and estradiol (76, 77). Across species, leptin and insulin have been shown to modulate reward processing *via* the dopamine system (34). Specifically, both leptin (78) and insulin (79) dampen dopaminergic signaling in (non-pregnant) rodents. Estradiol, progesterone, and glucocorticoid levels have been linked to cognitive functioning in both pregnant (80) and non-pregnant (for example, menopausal) (81) women. For instance, higher estradiol and lower cortisol levels have been associated with worse verbal recall ability in pregnant populations (82). Insulin resistance (83) and leptin resistance (84) have been associated with cognitive impairment (such as mild cognitive impairment) in non-pregnant populations, and insulin resistance in gestational diabetes mellitus may contribute to worse cognitive performance during pregnancy (85). For instance, pregnant women with gestational diabetes mellitus performed worse than non-diabetic pregnant women on the Montreal Cognitive Assessment test, which measures a range of cognitive functions (86). Although more work on these relationships in pregnancy is needed (80), initial evidence points to how these systems are interrelated. In the following sections, we will more closely explore how pregnancy-induced changes in reward processing and cognition may impact eating behavior.

## Reward processing

### Pregnancy and reward processing

Pregnancy alters reward processing in humans. Hoekzema et al. (87) found a reduction in gray matter volume in the right ventral striatum (a region important to reward processing) from pre- to post-pregnancy. Further, the striatum was more strongly activated when viewing images of one’s own vs. other baby, and the degree of this striatal activation was also associated with the degree of gray matter reduction (87). This has been interpreted as synaptic pruning representing an adaptive specialization, such that mothers’ reward processing areas are altered during pregnancy and show strong responsivity to infant cues (87). Such reward processing adaptations during pregnancy may predict responsiveness in domains other than infant-reward. Indeed, a neural reward signal in response to monetary reward during pregnancy predicts later self-reported bonding between mother and child (88). Research on reward processing in pregnancy is currently quite limited, but presents an exciting avenue for future research on dietary choice.

More research exists regarding the postpartum period, and these findings support our current understanding of the reward processing changes that occur in the transition to parenthood beginning in pregnancy. Gray matter volume change from early to later postpartum is associated with a positive perception of one’s baby (89), and reward processing of infant cues in postpartum women show strong activation in response to own-infant stimuli in reward-related areas including the nucleus accumbens (74). The role of reward processing in parenthood is also supported by the finding that dopamine receptor genes in human mothers have been associated with maternal behavior, for example, orienting to one’s infant (90). Pregnant and postpartum rodent models further highlight this important transition in reward processing, since rodents transition from finding pup cueing aversive to rewarding (75). Postpartum rodent mothers have greater activation in dopamine reward pathways in reaction to the suckling of their offspring than they do in reaction to cocaine exposure (91). Further, they will bar press for contact with pups (92). Additionally, agonists of D1 receptors [which are important in reward-related learning (93)] can facilitate maternal behavior in pregnancy-terminated rats (94). Much of this reward-related research has focused on the postpartum period and reward responses toward offspring, likely because this is most relevant to maternal behavior. However, it is important to remember that a substantial transition in reward-related brain areas occurs already during pregnancy in humans (87), and this carries important implications for eating behavior during pregnancy, as we will see in the following sections.

### Reward processing and eating behavior

Reward processing is one of the primary drivers of food consumption (34), and motivates consumption through the rewarding properties of the food rather than due to metabolic demand (95). Reward-related eating has been associated with excess food intake and higher body mass index (96). Reward motivations for eating consist of both liking and wanting motivations (97). Liking can be described as a positive, affective reaction to a food’s palatability, whereas wanting can be described as an incentive motivation to eat (97). Dopamine functioning, such as dopaminergic projections to the striatum, is especially important in wanting (98). For example, using lesions to eliminate dopamine in rats resulted in a lack of motivation to seek out or consume food, even though taste reactions remained the same (99). Further evidence for the role of dopaminergic functioning in eating behavior is that altered dopaminergic functioning has been implicated in obesity (100). Therefore, an event like pregnancy which alters such reward pathways may impact eating behavior through affecting reward-related eating.

### Pregnancy, reward processing, and eating behavior

Reward processing is a key mechanism of food intake and seems to be modulated during pregnancy. One of the rare studies investigating this question in humans is the Pregnancy Eating Attributes Study (101). Here, greater reward-related eating during pregnancy was associated with lower scores on the Healthy Eating Index (102). Further analyses showed that reward-related eating was associated with higher calorie consumption after satiety (103). Interestingly, questionnaire-based food reward measures (self-reports), however, did not correlate with excessive gestational weight gain (96). Changes in the processing of food reward in pregnant rodents have been reported, with a recent association being found between changes in dopaminergic signaling and craving-like eating episodes (104). Postpartum rats, alongside an increased preference for pup cueing, show an attenuation in preference for food cueing in a conditioned-place paradigm compared to virgin rats (105), suggesting a reprioritization of reward types.

Since reward processing plays a large role in guiding dietary decision-making, these changes to reward processing during pregnancy should be considered when attempting to promote healthy eating behavior. Future research could employ neuroimaging methods to better understand how changes to the structure of the striatum and to dopaminergic signaling during pregnancy impact reward-related eating.

## Cognition

### Changes to cognition during pregnancy

Pregnant women experience changes in cognitive functioning. A majority of pregnant women report cognitive impairment, often termed “pregnancy brain” (80). It has often been suggested that this may demonstrate a trade-off with gestation, parturition, and maternal behaviors (82, 106). Women may undergo some “cognitive reorganization” during pregnancy, with functions such as social cognition given precedence, and others, such as memory, given a lower priority (107). It could also be that the energy demands of the fetus may impact upon cognitive function (108). Structurally, there are overall decreases in gray matter volume in pregnancy (109, 110) in areas associated with cognitive functions like memory that may be altered during pregnancy, such as the hippocampus (109).

One domain of cognition impacted by pregnancy is executive functioning. This is a collection of higher order cognitive processes that are utilized when we act in a flexible, goal-oriented manner, and include inhibition, working memory, and flexibility (111). A recent meta-analysis by Davies et al. (112) found that executive functioning (including attention, planning, cognitive flexibility, and inhibition) significantly decreases in pregnant women in the third trimester. This supports a previous review that found that working memory seemed to be particularly impaired in pregnant women (113). Conversely, Fiterman and Raz (114) found that pregnant women have better inhibition in a behavioral task, and these findings were supported by event-related potential (ERP) neural signaling (114). Pregnant women’s response times were also slower (114), and these results suggest that pregnant women may be more cautious in their decision making. This finding is supported by a recent study by Chen et al. (115), in which pregnant women show higher risk aversion in the Columbia Card Sorting Task. Research so far suggests pregnancy may alter executive functioning, but work on this is limited (112). The direction of change remains unclear, but pregnancy may be associated with better inhibition.

A prevalent cognitive impairment reported during pregnancy is worsened memory (116). Overall, pregnancy appears to be associated with a decrease in memory function in both subjective reports and objective measurements. A meta-analysis by Davies et al. (112) found that memory (including working memory, long-term memory retrieval, and recognition) was broadly impacted by pregnancy, with a decrease in overall memory performance. This occurred during the third trimester in correlational studies, and the largest reduction in memory performance in longitudinal studies occurred between the first and second trimester (112). A previous meta-analysis by Henry and Rendell (113) found some measures of memory to be impacted by pregnancy, specifically free recall and delayed free recall, and the executive component of working memory. It is important to note that, while these findings are robust (112), the effects that have been found are small (113) and within normal ranges of cognitive functioning (112).

Despite small effect sizes, these memory impairments might impact the daily lives of pregnant women (117). Pregnant women report subjective memory impairment (118). Further, “naturalistic” measures of memory function, such as remembering to make a phone call or complete a time-logging task in the upcoming week found that pregnant women performed significantly worse than non-pregnant controls, even though they performed equally well on lab-based measures of memory function (113, 119), and this correlated with subjective impressions of memory function (119). Such studies shed important light on the ways in which cognitive impairment may affect the everyday lives of pregnant women, and an important domain that may be affected is eating behavior.

### Cognition and eating behavior

Executive functioning has been linked with dietary choice. This relationship depends both on the facet of executive functioning as well as the facet of eating behavior being considered. For example, initiation of healthy eating behaviors, vs. inhibition of unhealthy eating behaviors, may be affected by different processes within executive functioning (120). A study by Allom and Mullan (121) found better inhibition to predict lower unhealthy food intake (saturated fat) while updating in working memory was associated with initiation of healthy food intake (fruits and vegetables). Overall, lower inhibition and greater impulsivity have been associated with a greater risk of becoming overweight or obese (101, 120).

Memory plays an important role in eating behavior [see Higgs and Spetter (122) and Seitz et al. (123) for recent reviews]. Episodic recall is important in food consumption, and this has been demonstrated by the “meal recall” effect. Cueing participants to recall a recent meal is related to less food consumption compared to being cued to recall something else, such as a meal from longer ago (124). This finding has been replicated several times, although it can also be modulated by contextual factors, such as mood (125) or dietary disinhibition (126). Initial memory encoding also guides later food consumption, and an oft-replicated finding is that distracting participants while they eat leads to greater food consumption later (122). For example, watching television while snacking has been associated with more food consumed at a later meal, along with worse recall of the amount that they had snacked (127, 128). Further, better episodic recall is associated with less uncontrolled and emotional eating, and more strategic dieting, as well as a higher likelihood of avoiding fatty food consumption (129). Interestingly, hippocampal volume (a brain area important in memory functioning) has been repeatedly associated with diet-related outcomes, such as being overweight or obese (123). Different cognitive functions play an important role in eating behavior, therefore changes in these cognitive functions during pregnancy will impact eating behavior.

### Pregnancy-induced cognitive function changes and links to eating behavior

From the above-described findings, we can conclude that pregnancy-induced changes in cognitive functioning, such as in memory and executive functioning, are very likely to impact dietary choices. Though there is very limited research on executive functioning during pregnancy, some of the available evidence suggests that pregnant women may have slower response times and greater inhibition. Future research could determine if this may positively impact dietary choice, since better inhibition may limit unhealthy food intake. Conversely, worse memory in pregnancy may exacerbate excessive food intake, since memory function has been linked to the regulation of eating behavior. It would also be interesting to better understand the possible relationship between a reduction of hippocampal volume during pregnancy, and the association between reduced hippocampal volume and a higher likelihood of being overweight or obese in non-pregnant populations. A better understanding of these relationships would allow us to work toward optimizing dietary advice and interventions during this critical period, by tailoring such approaches to both counteract cognitive impairment, and harness cognitive changes that could promote healthy eating behavior.

## Conclusion and future perspectives

This review has considered pregnancy-induced changes to hormonal, metabolic, reward-related, and cognitive processes guiding dietary choice. As outlined above, some changes, such as metabolic and memory changes, may make it difficult to make healthy dietary decisions during pregnancy, since the pregnant body is adaptively geared for weight gain, and impaired memory may negatively impact eating behaviors. Other changes, such as those related to reward processing and inhibition, may encourage beneficial eating behavior, since pregnant women may be more attuned to offspring-related reward (including the health of the baby), and greater inhibition could encourage healthy food choice. Future research is needed to further investigate the influence of these changing mechanisms on dietary intake.

Despite limitations in terms of research available in this area, we are already able to integrate these findings from different fields to produce tangible suggestions for the improvement of dietary behavior in pregnant women. For example, we have seen that memory impairment negatively impacts healthy food choices, and that memory is impaired in pregnancy. Therefore, practices aimed at improving memory of food consumption, such as food journaling (122), may be especially helpful during this time period. Further, since reward responsiveness to offspring develops during pregnancy, it may be helpful to increase education regarding the impact of nutrition on offspring outcomes. Currently, nutrition education for pregnant women is inadequate (130). If it were improved, pregnant women would be more aware of health information which may increase the reward value of healthy food once it has been explicitly connected to offspring wellbeing.

Effective promotion of healthy nutrition during pregnancy could be implemented both through healthcare providers as well as *via* digital devices. Healthcare providers such as physicians, midwives, and counselors could educate pregnant women on the reward-related and cognitive processes guiding dietary choice, and implement practices that target the pregnancy-induced changes in such processes. A more cost-efficient strategy could involve current technology such as mHealth (the use of mobile devices in healthcare) that have wide availability (131). Current mobile health interventions for pregnancy have shown only limited success (132), suggesting room for improvement. Mobile interventions for pregnancy could be improved by integrating practices targeted at the pregnancy-induced changes discussed in this review. For example, smartphone interventions can effectively improve memory function in the context of food consumption in non-pregnant populations, through, for example, recording meals, and this can lead to increased reported awareness of food consumption and weight loss (133). Another mobile intervention altered reward value of food through increasing awareness with mindfulness practices in a non-pregnant population (134). Such a practice could be tailored to the changes women experience in reward processing in pregnancy by promoting awareness of the health associations of their dietary choices for their offspring, as this could alter food reward value for pregnant women in a manner encouraging healthy food choices. Understanding pregnancy-induced changes to hormonal, metabolic, reward-related, and cognitive processes would provide evidence for which tasks, training, and educational materials would be most effective in promoting healthy eating behavior in pregnant women, both from healthcare providers and from digital sources.

A limitation of this review is the limited research in this domain, and therefore our use of postpartum studies. However, from what we know of the dramatic hormonal and anatomical changes occurring during pregnancy, we can assume that postpartum findings have something valuable to offer in helping us better understand changes in pregnancy. Another limitation is that much of the research currently available employs rodent models. While these are helpful in gaining an understanding of how we might expect pregnancy to affect women, it is important to verify such findings in humans.

In conclusion, a better understanding of pregnancy-induced changes to hormonal, metabolic, reward-related, and cognitive changes would provide actionable suggestions for improving important health outcomes for pregnant women. More research in this domain is essential because dietary choice during pregnancy affects both short- and long-term outcomes for mother and child, such as cardiovascular and metabolic health. The health consequences of dietary choice during this period extend throughout the lifetime, carrying significant personal and financial implications, making this an important research area to pursue.

## Author contributions

TW and SP: conceptualization, investigation, methodology, formal analysis, writing, and project administration. TW: visualization. SP: supervision and funding acquisition. Both authors have read and agreed to the published version of the manuscript.
